# Remarks on the Operation of Excision of Hip and Knee Joints

**Published:** 1884-10

**Authors:** Chr. Fenger

**Affiliations:** Chicago, Surgeon to Cook County Hospital; Prof. of Pathology, Chicago Medical College. Member American Surgical Association; 214 East Ohio Street, Chicago


THE CHICAGO

### MEDICAL JOURNAL AND EXAMINER.

VOL. XLIX. OCTOBER, 1884. No. 4.

### ORIGINAL COMMUNICATIONS.

### ARTICLE I.

REMARKS ON THE OPERATION OF EXCISION OF HIP AND KNEE JOINTS, WITH EXHIBITION OF PATIENTS. *By* CHR. FENGER, *Chicago, Surgeon to Cook County Hospital; Prof. of Pathology, Chicago Medical College. Member American Surgical Association.*

[*Read before the Illinois State Medical Society*, 1884.)

*Gentlemen:* I wish to present to the Society this afternoon a few patients on whom I performed, within the the past four years, excisions either of the hip or of the knee joint. In doing so, it cannot be my intention to discuss fully the subject of these operations; it is too comprehensive a one to be illustrated by a limited number of cases.

During the past ten years our knowledge of articular diseases has been greatly enriched by pathological research, and, at the same time, the practice of joint-surgery has undergone some modifications since the introduction of the antiseptic method. How far our expectations of the success of this method have been realized, is a matter which admits of discussion. The cases which I wish to exhibit here are, of course, only supposed to throw a little light on a few points in this great question.

Let me first call your attention to the hip joint. It is hardly necessary to point out to you that the old name, morbus coxarius, or caries of the hip joint, is altogether too vague a term to be of any service, even to the practical surgeon. Suppurating synovitis of the hip, osteo-myelitis of the head and neck of the femur, and the tuberculosis of the hip joint, are to be distinguished from each other, not merely because pathology shows them to be different diseases, but because these different diseases, if they are to be treated rationally, demand different modes of treatment.

By far the greater number of cases of morbus coxarius are cases of tuberculosis. It is still an open question whether the tuberculosis originates as a local osteo-tuberculosis in the head or neck of the femur, or in the acetabulum, as Volkmann believes it to do in most cases, or whether it may not primarily be a tuberculous synovitis. But be this as it may, the knowledge of the fact that a primary osteo-tuberculosis often exists a long time without giving any symptoms (of hip disease), until by opening into joint, it causes a tuberculous destruction of the latter, has already had its beneficial application. Timely operations on the great trochanter have, by removing the local osteo-tuberculous focus, saved a number of joints from total destruction. As the hip joint is covered by a thick mass of soft parts, it is materially much less accessible than the knee joint. In hip cases an early diagnosis and treatment, by which the joint may be saved, will probably always remain exceptional. There is hardly a question in practical surgery which is more difficult to decide than whether and when, in a given case, a tuberculous hip joint should be excised. Statistical tables, showing the mortality or the duration of the disease, teach us absolutely nothing in regard to this point, as in them mild and severe cases are promiscuously thrown together. Taylor gives a mortality of 12½ per cent., in 288 cases, treated without operation, 9 per cent. out of the twelve died of exhaustion and amyloid degeneration of various organs. Now, there is nothing in his tables to render it improbable that judicious operations would, in this instance, have depressed the death rate to a still lower percentage, that they would have saved some of the 9 per cent. who died of exhaustion and amyloid degeneration. Lessing's excision statistics show a mortality of 64 per cent. But, as no distinction is made between early and late operations, they are far from proving that 36 per cent. of otherwise fatal cases of hip-joint were saved by the operation.

In early excisions of the hip, that is, in those performed as soon as an abscess communicating with the joint has formed, and before the patient has become emaciated and exhausted by long-standing fever, the results of the operation are, so far as life is concerned, favorable enough. But we cannot do away with this objection to early excisions, that by them a healthy neck of the femur and trochanter are removed, which, if the abscess had been opened with antiseptic precautions, might have been saved, and might have given the patient a more useful limb. On the other hand, if we wait till one or more abscesses have either broken or been opened with the knife, till, from failure of carefully and constantly applying antiseptic dressings to the discharging fistulas an exhausting fever, which in reality always means pyæmia or septicæmia, has established itself and reduced the patient's strength to a degree dangerous to life,—if, I say, we wait till all this has taken place, then we run the risk of operating too late. The septicæmia will probably progress in spite of the operation, and the patient will die. By an early operation his life would probably have been saved. We are rendered unwilling to operate early, chiefly by consideration of the fact, that in a great number of cases, where one or more abscesses had formed and been opened and running fistulæ remained for a long time, the latter finally closed up spontaneously, and the patients recovered, and this not so very rarely with a useful and even to a certain extent movable joint. Out of Gibney's 80 cases of spontaneous recovering, this occurred in 48.

In 1873, Volkmann, whom I consider to be the greatest now living authority on joint diseases, gave four indications for excision of the hip in cases of tuberculosis.

(1) The presence of abscesses and fistulæ with copious suppuration, together with a rise in temperature, which indicates that patient's strength is beginning to be undermined.

(2) High fever from acute suppuration in the articular capsule, coming on suddenly in the course of a chronic case.

(3) An iliac abscess, which shows that the acetabulum has been perforated.

(4) Established suppuration with dislocation of the head of the femur backwards, so that it can be felt behind the acetabulum, covered by the gluteal muscles.

I think it safe to add a fifth indication—

(5) Detachment of the epiphysis of the head of the femur and its lying in the articular capsule as a large sequestrum.

When Köster, Volkmann, Schüppel and others discovered, in the course of their investigations, that fungoid arthritis or white swelling, and, consequently, the common form of morbus coxarius, was a local tuberculosis, this as such was for a time also looked upon as an indication for excision of the hip joint.

It was then thought that nothing short of an early removal of the miliary tubercles from the bone, the capsule and the walls of the abscess cavities (if abscesses had already formed) could prevent a general and fatal tuberculous infection from spreading from the primary focus. It so happened that about the same time Lister gave his antiseptic method to the profession, and, as early operations were by this method deprived of their former danger to life, a number of early excisions of the hip were made. Six or eight years ago it was generally deemed justifiable to operate at the time of the formation of the first abscess, if possible before it had opened. Surgeons had become hopeful. Lister's method seemed to secure here, as it does in other cases, healing by first intention, and the operation would eradicate the tuberculosis. Besides, matters seemed very much simplified as to recognizing the right moment when an operation becomes imperative, and expectant treatment is no longer permissible. Unfortunately, however, we were in this respect soon thrown into the old difficulties. It was found by all operators, I believe, but first expressly stated by König (in Langenbeck's Archive, 1880), that healing by first intention, *i. e.* healing in four to eight weeks, still remained a very rare exception. An apparent healing would take place, but after a while new crops of miliary tubercles would grow up in the canals made by the drainage tubes, or, in cases where the openings had closed, in the cicatrices left by them; new abscesses and ulcers would form. Moreover, it was learned that, notwithstanding early operations, about the usual twenty per cent. of these patients would, sooner or later, even after apparent cessation of the local process, succumb to tuberculosis of one or more of the internal organs. As pathology now began to rob this disease of some of its terrors, by demonstrating that, when local, it not so infrequently ended in recovery, pathological researches concurred with surgical experience in bringing us back to our former more conservative standpoint regarding indications for excisions of the hip. We again shrink from the operation before patient's life is threatened with danger, and are loth to take away living bone, which, after the termination of the disease, might help to form a solid anchylosis, or even a somewhat movable joint.

All of us, however, who have lived through this interesting period in the history of articular surgery, will remember with pleasure, that, in the great majority of these cases of early excisions, the after-treatment was much shorter than it had formerly ever been; the operation promptly and decidedly relieved the patient of his sufferings; the fever disappeared, and the malposition was corrected in a much shorter time than could ever have been done by conservative treatment. Unfortunately we do not know whether the limbs resulting in the end from these operations were the best that could be obtained under the circumstances, or whether their function would not have been improved if the conservative treatment had been employed. We must confess that with regard to the whole question we are somewhat at sea. The final results of early operations and of conservative treatment are not sufficiently different to compel us to make one or the other the invariable rule in the treatment of tuberculous hip disease. In a given case we will, of course, always prefer conservative treatment, if by it we expect to obtain as good a result as by an operation.

Allow me first to present to you a few cases in which early operations were performed.

*Case 1.*—Edward Prout, eight years old, was admitted to the Cook County Hospital on August 5, 1880. His parents were living, and both of them in good health. A brother of his was at the time suffering from a tuberculous inflammation of a testicle, following gonorrhœa. Patient had always been in good health till three years and a half ago, when he fell from a swing, his right knee striking on a stone. About one month later he began to complain of pain in the knee. His right leg soon acquired a lameness which would come and go during the following year; the pain in the knee remained rather constant. About one year after receiving the injury he had a hip-support put on by his physician, and extension made. His pain was thereby somewhat relieved. Patient wore the apparatus about three months; it was then taken off, as he was unable to pay for it. Patient was now admitted to St. Luke's Hospital, and was thence, in the summer of 1878, taken to the Mercy Hospital. About one year later an abscess formed in Scarpa's triangle; an opening was made at the upper and inner part of the thigh, and the pus was evacuated; a sinus remained, which was still open at the time of patient's admission to the County Hospital. A short time after the formation of this abscess a second one formed at the upper part of the posterior aspect of the thigh. This abscess, like the first one, was opened, and left a fistula. In the beginning of 1880 patient had apparently recovered. The affected limb had become about 1½ to 2 inches shorter than the sound one, but wearing a high-soled shoe on the foot of the diseased leg, he was able to walk on it. This condition continued up to the beginning of July, 1880, when patient was kicked by a boy in the upper and posterior part of his right thigh, where a swelling, *i. e.*, an abscess, soon formed below the great trochanter. This abscess was opened, and a discharging sinus remained. Patient was now no longer able to walk; he complained of more or less pain all the time, and the three sinuses kept up a constant discharge; the thigh gradually became flexed.

An examination showed the boy to be rather pale and slim; there was no rise in temperature, his pulse was a little accelerated, between 90° and 100°. His lungs, heart and urine were found to be normal. He could walk only with crutches, as he was prevented from putting his right foot to the ground by the adduction and flexion of his hip. There was one and one-half inches shortening. The sinuses on the internal, posterior and external aspects of the thigh discharged a moderate amount of pus. A probe introduced into the external sinus passed up behind the neck of the femur into the joint where roughened bone was felt. Passive motion caused pain in the joint.

On August 20, 1880, an excision of the hip was made, as follows: I made an incision anteriorly along the anterior sinus (Langenbeck's incision), with a view to remove the head of the femur only; but before getting into the joint a considerable venous hæmorrhage occurred, the source of which was probably in the bulbous enlargement formed by the vena saphena magna just before joining the femoral vein. As it was impossible to stop this hæmorrhage in the dense cicatricial tissue of the sinus, otherwise than by ligature en masse, I had to give up the anterior incision. I therefore made the straight external incision over the outer side of the trochanter major, below which I sawed off the bone. As I operated subperiosteally, the cartilaginous portion of the trochanter major was left connected with the periosteum. The head of the femur had disappeared entirely, and the roughened end of the neck was found above and back of the acetabulum, which was covered with a thick, soft mass of tuberculous granulation tissue. I scraped the acetabulum which was not perforated, with a gauge and the sharp spoon; the walls of the sinuses were likewise scraped. Drainage tubes were then inserted in the three sinuses, and made to run up into the acetabulum. The lips of the wound were then united by sutures, and a Lister's dressing applied. The limb was placed in an abducted position, and a Verity's splint put on, and extension made by means of a piece of rubber tubing and strips of adhesive plaster.

Aug. 21. During the after-treatment the evening temperature remained steady, in the neighborhood of 100°; only once did it rise to 100.5°. Patient was free from pain, and commenced to eat towards the end of the first week. The wound, which healed by first intention and without suppuration, was dressed twice weekly. From the beginning of the third week the drainage tubes were shortened every time the patient was dressed.

Sept. 16. Patient was able to draw his knee up a little.

Sept. 18. The last drainage tube was removed.

Sept. 20. *i. e.*, four weeks after the operation, the wound had healed completely.

Sept. 25. The splint was taken off.

Patient was kept in bed two weeks longer; a high sole was placed under the foot of the sound limb, and he was allowed to walk with crutches.

A year ago an abscess formed behind the trochanter, which was opened, and then closed up in a few weeks.

He is now well nourished, able to bear the whole weight of his body on the affected limb. There is little mobility in the newly-formed joint, about 20° flexion, no ab- or adduction nor rotation.

*Case 2.*—Fred. Hunneman, a child of healthy parents, began to limp when 3½ years old. Extension by weight and pulley was tried during the first year of the disease. In its third year an abscess formed below the great trochanter; there was considerable pain in the joint, and he had some fever; his general health was impaired, and he emaciated.

The leg was flexed and adducted. Three and one-half years after the commencement of the disease I made an incision of the hip, in February, 1881. After the operation the patient lay in bed eleven weeks, splinted and in extension. In six weeks the wound had healed up to the holes for the drainage tubes. In fourteen weeks he was allowed to be up and about, having a plaster-cast on the limb. There had been no fever, nor drain after the operation, and his general health gradually improved. One year later, in January, 1882, an abscess formed below the gluteus maximus. On opening it, it was found to be an intra-pelvic abscess, which had formed subsequent to the perforation of the acetabulum. It had made its way out of the pelvis through the great sciatic notch along the pyriformis muscle. The walls of the abscess, within and without the pelvis, and the pelvic surface of the sciatic bone, were scraped, (with a sharp spoon). The wound healed in four to five weeks, a small fistula only remaining. Half a year later, in August, 1882, all fistulæ had healed up definitively. Since that time the patient has walked about with crutches, having a high sole under the foot of the sound limb. He is in excellent health. When allowed to try, it is seen that the affected limb is capable of bearing considerable weight.

*Case 3.*—John Prince. In the fall of 1877 he began to feel pain in his right knee, and later on, in the thigh and groin. There had been no traumatic cause for the coming of this pain, which gradually became worse. He was disabled from walking without a cane, and from sitting down, as he could not flex his thigh sufficiently. About Christmas, 1877, he fell on a stone-sidewalk, striking on his hip, and the pain increased the following day. He then lay in bed, with extension on, five months, but his condition remained about the same. On being so advised, he tried a portable extension apparatus, something like Taylor's machine. Four months subsequently an abscess formed on the outer aspect of the thigh; it was opened, and left a fistula. In January, 1879, he entered the Cook County Hospital, was put to bed and kept in extension for four months; he then got up and walked about with crutches and a high sole under the foot of the other leg. During the following three months the condition remained as it was, the fistula discharging considerably and tenderness existing around the joint. In September, 1879, I excised the joint below the lesser trochanter. During the fall and winter patient had two attacks of erisypelas. He got up nine months subsequent to the operation, two years to his entering the hospital and three and one-half years to the beginning of the disease. Since that time he has steadily improved. He has since picked up so much flesh, that he is now almost twice as heavy as he ever was previous to or during his stay at the hospital. He walks easily with a cane; can bear the whole weight of the body on the limb operated upon. There is considerable active mobility in the new-formed joint, flexion 90°, abduction and adduction 15°, and considerable rotation.

In looking upon these as early operations, we must bear in mind that discharging fistulæ, abscesses, and, in two of the cases, some dislocation of the head of the femur already existed. In the Hunneman case we were dealing with an acetabular coxitis, as was shown by the later formation of a pelvic abscess, which was scraped out and finally healed up. In this case, especially, the pain and fever were promptly relieved by the operation, and from the very day of its performance, the local disease, as well as the general health of the patient, improved uninterruptedly.

A very extensive tuberculosis of the whole upper third of the femur induced me to make an excision of the hip in the following case:

*Case 4.*—Thomas Hughes, aged 16, was admitted to the Cook County Hospital, September 25, 1882. His parents were healthy; a brother of his, like himself, suffered from hip disease. *Four years previous* to his admission he began to feel some pain in his hip, groin and thigh, and he acquired a slight limp. About the same time an abscess appeared on the dorssum of the foot; it was opened, and a chronic swelling remained, which finally led to the removal of the tuberculous first metatarsal bone. About one year later, patient fell, his hip striking on the floor. A swelling soon appeared in the neighborhood of the hip, and patient complained of pain in the hip and in the knee. Several abscesses formed, and were opened; sinuses remained, which alternately closed and opened, discharging at times considerable quantities of pus. Three months before patient's admission, three small pieces of bone were taken out of a fistulous opening in the groin.

On examination, the leg was found to be flexed and adducted; as there was three inches shortening, the foot did not reach the ground. The region of the hip was rounded and enlarged, and it presented one sinus anteriorly and three on the outer side. Passing the probe along these sinuses, I would feel roughened bone in the hip joint, as well as on the trochanter major and the upper part of the shaft. The foot was deformed, as the removal of the first metatarsal bone had produced a retraction of the great toe.

On September 28, I excised the joint. The incision over the trochanter had to be prolonged downwards over the upper third of the shaft of the femur. The head, trochanter, and 2½ inches of the shaft of the femur had to be removed. On examining the (upper) sawed end of the remaining shaft, it was found thickened, and containing a tuberculous cavity. This was gouged out, and the diseased bone had to be chiseled away for a distance of 1½ inches, in order to reach healthy bony tissue. The preserved specimen shows considerable periosteal tuberculosis, with irregular periosteal exostoses, in which may be seen, here and there, the rounded cavities characteristic of local osteo-tuberculosis. I scraped the acetabulum and the walls of the existing sinuses, put in a sufficient number of large drainage-tubes, dusted the wound with iodoform and closed it. A Lister's dressing was applied, the limb placed in a Verity's splint, and extension made.

Patient had been weak during the operation, and remained so for some time. There was considerable suppuration during the first month following the operation. The pulse rate varied between 110 and 130, but the temperature never rose much above normal.

Oct. 13. A bed-sore formed over the sacrum, which grew larger for a couple of weeks; later on, bed-sores formed over the spinous processes of the lumbar vertebræ.

Dec. 4. The abdomen was greatly distended, as were also the superficial veins. Percussion showed the spleen to be enlarged considerably, and the dullness of the liver extended six inches below the ribs. (Amyloid spleen and liver.) The urine was clear and acid, and no albumen was detected in it.

Jan. 21. All bed-sores had healed.

Feb. 1. The splint was removed and patient was allowed to sit up and to walk about with crutches, wearing a high sole under the foot of the sound limb.

March 21. Patient was dismissed from the hospital, with an order to present himself once a week to have his limb dressed.

Patient is now robust, of healthy appearance; the fistulæ have been closed for half a year; he can bear the weight of his body on the limb operated upon; has active flexion of about 70°; some ab- and adduction and some rotation.

The extent of active mobility patient has got in his hip is remarkable, considering the amount of bone removed. He can lift his leg and flex the thigh almost to a rectangle (to an angle of almost 90°.) This would be impossible if not a considerable reproduction of the bone had taken place, not only of the removed part of the shaft of the femur, but also, to some extent, of the great trochanter; for flexion is effected by the ilio-psoas muscle.

In cases where the epiphysis of the head of the femur is detached and lies as a sequestrum in the acetabulum or in the abscess cavity surrounding the neck of the femur, there can be no doubt as to the propriety of an operation. However, in a given case of chronic hip disease, the diagnosis of this detachment can, as a rule, not be made. There are no objective symptoms (so to speak) peculiar to this condition. As the detached epiphysis lies either in the acetabulum or is covered by a thick layer of soft parts, it cannot be felt. Unless the history of the case, together with the age of the patient, point toward such a condition—*e. g.*, acute suppurative inflammation (of the joint) immediately following a fall on the great trochanter, or an acute suppurating osteo-myelitis of the upper epiphysis of the femur—the detachment will probably not be detected till the excision produces the necrotic head of the femur. This detachment is rarely met with in tuberculosis. An instance of this kind is the following case:

*Case 5.*—Benjamin Cleaves, æt. 13, was admitted to the Cook County Hospital Aug. 21, 1883. There had been no consumption in his family, and he himself had always been healthy till, during the latter part of January, 1883, his left hip began to ache a little. As the inconvenience was slight, he paid no attention to it. One evening, while lying in bed, the pain became troublesome, and, in attempting to rise, he suddenly felt a sharp pang in the groin. The pain would return again and again, till it finally became almost continuous. Three weeks after the commencement of the pain, patient had become obliged to pass most of his time sitting quietly in a chair. In March, a physician was called, who advised the boy's parents to put him out into the country. In June, an abscess on the outer side of the thigh was evacuated, and from the opening made pus continued to be discharged. No antiseptic dressings were applied. In July, patient returned to the city, and I saw him in the beginning of August. I found him lying in bed, and very much emaciated; pulse 124; temperature 101.7°. The right thigh was adducted and flexed; the slightest movement caused great pain in the hip and in the knee. The knee joint was normal. During the night he would wake up and complain of severe pain in the hip. The region of the joint was rounded, swollen and very tender to the touch. On the outer side of the thigh, three inches below the great trochanter, a fistulous opening discharged a considerable quantity of matter. Patient stated that he had never injured his hip, except two years ago, when, running along a stone wall, he struck against it with his hip, producing an abrasion of the skin. After this abrasion had healed, he felt no pain for a year and a half afterwards. The urine was normal.

Aug. 23.—Excision of the hip. I made the usual straight incision over the great trochanter. A large cavity filled with pus and tuberculous granulative tissue was found to have formed around the great trochanter and the neck of the femur, which were to be removed. The necrosed epiphysis of the head of the femur was found detached in this cavity. The cartilage of the great trochanter was not removed. The walls of the cavity, the acetubulum and the sinuses were scraped. It was all dusted over with iodoform and sufficient drainage provided for by several button-hole openings, posterior laterally and external to the joint. An antiseptic dressing and a Verity's splint were put on, and extension made.

The wound discharged profusely. The evening temperature never sank below 101° or 102°; pulse always 120 or more. New pocketings of pus took place below the gluteal muscles. Bed-sores formed over the sacrum. In December, pus was detected in the pelvis, burrowing along the iliacus muscle. A little later an incision was made in the lumbar region and a drainage-tube inserted. The injected fluid ran out at the opening in the thigh below Poupart's ligament. This large suppurating tract kept on discharging a great quantity of matter. Patient gradually lost strength, and died of pyæmia, Jan. 8, 1884.

His case might be called one of early operation, if only the time from the beginning of the disease were to be considered. But the operation was made necessary by the profuse suppuration and septic fever, which, continuing in spite of the operation, caused the patient's death. Pyæmia set in in this case, as no antiseptic precautions whatever had been observed at the opening and during the after-treatment of the abscess. In this connection I should like to mention a point, regarding the influence the antiseptic method has had on the course of our treatment of this disease. *It is of the utmost importance, and should never be forgotten.* Just as the antiseptic method permitted us, at least for a time, to operate early, as soon as the first abscess had formed, because by it the operation had been deprived of its great dangers to patient's life, in the same way has the antiseptic method made it possible quietly to wait till the time for the late operation has come, without imperiling thereby the life of our patient, as was done formerly. The history of cold, that is, tuberculous abscesses, before and after the introduction of Lister's method, is too well known to require much discussion. It is true that, in former times, opening of a cold abscess was not invariably followed by septicæmia. But it is also true that formerly opening of an abscess was always done at the risk of patient's life, and no surgeon could avoid the danger. (Now the patient's life is, in this respect, in the surgeon's hands; if a thoroughly antiseptic dressing be applied and kept over the opened abscess, we know that septicæmia will not set in.)

Late operations, aside from those cases which have progressed too far to be benefited by the surgeon, are preferable, as we ourselves are concerned, for we need not trouble our minds with the question, whether excision had become a necessity or not. As regards the patient, he will, as a rule, have to go through a course of protracted and troublesome after-treatment; the limb may become unduly weakened, either from lack of growth on account of inactivity, or from secondary changes due to chronic inflammation, or atrophy of the bones of the hip joint, of the ilium as well as the head of the femur.

In illustration of these two points, I wish to present the following cases:

*Case 6.*—Louis Anderson, aged 19, cigar-maker, was admitted to the hospital Sept. 1, 1882. He had suffered for ten years from morbus coxarius of the right hip. A year or two after the beginning of the disease an abscess opened on the outer side of the joint; about four years later it closed again; it was followed by a second and third abscess which opened anteriorly and posteriorly and left discharging sinuses. Pain, shortening and malposition rendered the limb entirely useless. On examining the patient I found the whole right lower extremity atrophied and much smaller than the other on account of retarded growth. Measurement showed the shortening to

2

amount to 3½ inches: the limb was adducted and flexed; the trochanter was felt an inch and a half above Nelaton's line; but very little passive mobility remained. Pressure on the anterior part of the hip was painful. The knee presented a genu valgum or bow-leg, and the head of the fibula was apparently enlarged.

Sept. 21.—The excision was made through the lower part of the great trochanter. The head of the femur and part of its neck had disappeared. A large abscess was found below the gluteus maximus posterior to the acetabulum; it was opened and its tuberculous walls were removed with the sharp spoon. The wound was closed and dressed in the same manner as in the above cases. During the first month of the after-treatment there was considerable discharge. The evening temperature would rise to 101° or 102° for a few weeks, but then it fell to normal.

Dec. 13.—The drainage-tubes were removed. A large portion of the wound had not healed, but formed an open tuberculous ulcer.

About Jan. 20, 1883, patient got an attack of erysipelas, which spread over the whole right leg. It subsided in two weeks.

Feb. 18.—A peri-articular abscess, situated on the inner side of the knee, was opened.

In the beginning of April, patient had a second attack of erysipelas, which lasted about one week. After patient had been in the hospital one year, he was dischaged, as he was able to walk about with the aid of crutches and a high sole under the foot of the sound leg. He still had large, granulating tuberculous sinuses.

The large granulating sinuses still remain and will have to undergo further treatment of scraping and iodoform dressing.

He can bear the weight of his body on the affected limb; there is only slight active mobility in the new-formed joint.

In this case the five or six years' duration of the disease had caused the growth of the whole extremity to be considerably retarded; besides, it had brought about a deformity of the knee, which in no small degree lessens the usefulness of the limb, and will probably do so for life.

The further disadvantages of a very late operation are well illustrated by

*Case 7.* Sina Anderson, aged 21, single, daughter of a Nebraska farmer, was sent to the Cook County Hospital by me, and was admitted June 8, 1882. She was of a healthy family, and had herself been a healthy child. In her thirteenth year, eight years before the operation, she was suddenly seized with an acute bilateral inflammation of the hip-joints, which was mild on the right, but severe on the left side. The right hip recovered completely within half a year, but in the left hip the inflammation terminated in an abscess, which was opened half a year after the commencement of the disease, and which had discharged pus ever since. Two years and a half before her admission to the hospital another abscess which had formed was opened, and a second fistula remained. In the beginning of the disease she had been confined to her bed for one and one-half years; after that time she could walk about with the aid of crutches. On account of the adduction and flexion of the thigh, which produced an apparent shortening of over three inches, she was at no time during those eight years able to use the affected limb in walking, or to place its foot on the ground. During the whole period pain and discharge would both come and go. She was obliged to sit in a chair all day, as walking with crutches was very painful. On examination she was found to be well nourished, *i. e.*, not emaciated, but anæmic. When standing, supported by crutches, she was unable to touch the ground with the toes of her left foot. The flexion and adduction of the affected limb had brought the left knee in a position anterior to and a little above the lower third of the right femur. The thigh and calf were atrophied, the latter was between one and two inches less in circumference than the calf of the sound leg. The soft tissues in the region of the hip had become hard from infiltration, and there was considerable enlargement and deformity. There was no active mobility in the joint; the slight passive movements which could be made, produced severe pain. Three sinuses discharged from the thigh; one opened on the inner side, at the lower angle of Scarpa's triangle; another on the same level on the posterior side, and the third one on the outer side, below and behind the trochanter major. Every one of these three sinuses led the probe up in the direction of the joint, which, however, could not be reached. A fourth sinus opened in the lumbar region, just above the crest of the ilium. The probe passed down in the direction of the joint, but only for a short distance. An examination through the rectum revealed neither thickening of the os pubis nor of the ischium. Pressure on the abdomen just above Poupart's ligament showed considerable thickening of the crest of the ilium, near its anterior superior spine, and also of the ilium on its pelvic surface. This pressure, as well as all pressure in the neighborhood of the hip, was very painful.

Heart and lungs were normal, the liver of normal size, and the spleen not perceptibly enlarged. The urine was straw-colored, clear, acid, and contained neither albumen nor sugar. The diagnosis was morbus coxarius consequent upon acute osteo-myelitis of the neck of the femur and suppurating synovitis.

Jan. 20. Pulse and temperature normal. Once in a while, however, the temperature would rise to 100°.

Jan. 21. Operation: Assisted by Drs. Jacobson and Lee, and the surgical house staff of the hospital, I made an incision ten inches long, in the line over the greater trochanter. I cut through the thickened soft tissues to a depth of over two inches and a half and then reached a cavity, filled with pus and containing the detached epiphysis of the head of the femur. It was of normal shape, denuded of its cartilage, and the articular surface was superficially corroded near its border. The neck of the femur and the greater trochanter were still visible but softened by fatty medullary atrophy to such an extent as to leave only a shell of bone as thin as paper, surrounding the yellow, fatty, butter-like tissue of the marrow. The bone was sawed off below and close to the lesser trochanter. Here, also, there was only a thin shell of compact osseous substance left. After removal of the head of the femur and of the trochanter the leg could be abducted and extended. The pelvis was not touched; no sinuses were seen leading into the ilium. The four sinuses were now scraped and each one received a drainage-tube. The cavity and the wound were dusted over with iodoform, the edges of the wound sewed together, and a Lister's dressing, a Verity's splint applied and elastic extension made.

The course of the after-treatment was normal, patient's appetite fair, and scarcely any rise in temperature till Oct. 25, when it rose to 103°.

Oct. 25. The wound was re-opened and about two ounces of pus were evacuated from the old abscess cavity where the head of the femur had lain. The walls of the abscess cavity were scraped and a counter-opening was made through the gluteus maximus. Thereby the temperature was brought down again, and the course of the treatment ran on normally till Nov. 23, when a rise in temperature to 104° announced an attack of erysipelas. This lasted a couple of weeks, and left several small subcutaneous abscesses in the leg, which healed up toward the end of the year.

Jan., 1883. In January an abscess was opened on the outer side of the hip.

March 11. The suspensory splint was removed.

April 22. Extension was put on with a view to abduct the leg.

June 3. One of the sinuses closed.

June 18. Patient was anæsthetized and the still open sinuses scraped with the sharp spoon. Several small fragments of necrosed bone were removed.

Aug. 15. The last fistula closed.

In September she was allowed to walk about supported by crutches and wearing a high sole under the foot of the healthy leg.

Oct. 10. She left the hospital for a private residence in the city.

1884. During the winter the sinus which had opened on the outer side of the hip and which had led up to the original abscess cavity, re-opened several times, discharged a little pus for a week or two and then closed again. The leg gradually grew stronger. When lying in bed she could now (in April, 1884,) raise the limb or draw up the knee to an angle of 20–30°; when resting on the sound foot, she had, in the hip joint operated upon, 30° of flexion, 10° of abduction and 10° of adduction. The leg which had become shortened by 2½ inches was in good position; it could bear the weight of the body when she was allowed to try. She was well nourished and not so pale as before the operation. The thickening of the soft parts around the hip was gradually subsiding; the thickening of the anterior part of the crest of the ilium, as well as that of the bone in the iliac fossa, is the same as before the operation.

She left the city with an apparatus somewhat like Taylor's, but provided with a flexible knee-joint. This apparatus enables her to walk comfortably with the aid of crutches.

In this instance the detachment of the epiphysis of the head of the femur could be suspected from the very beginning of the disease. The history provided sufficiently clear evidence, not of tuberculosis, but of suppurating coxitis, which would probably be osteo-myelitis. Whenever, in a child, in the course of this disease, suppuration sets in, and continues with the formation of multiple abscesses, detachment of the epiphysis may always be inferred, and excision is indicated. If an early operation had been performed in this case, it is more than likely that no chronic osteo-periostitis of the ilium would have occurred, and patient, besides being spared many years of suffering, would have recovered much more speedily after the operation.

Just here, I should like to insert a few words concerning a peculiar form of atrophy of bone, which is found in some cases where there is long-standing suppuration in close proximity to osseous tissue. We should be well acquainted with the matter, as it is of no mean practical importance at the time of operating. In this peculiar form of atrophy, which is especially met with in the near neighborhood of suppurating joints, the osseous substance, cancellous as well as compact, is absorbed, and its space is occupied by a fatty infiltrated marrow. This marrow is poor in vessels, of soft consistency, and bright yellow in color; it somewhat resembles butter. The bone proper is reduced to a mere shell, here and there as thin as paper, and, on account of its thinness, of an elastic feel. Such bone is, of course, useless. But it is very important to remember that this kind of atrophy is altogether unlike an inflammatory, destructive process. This medullary tissue of the bones, whether young or old, red, or yellow and fatty, is always capable of producing new osseous substance. That formation of new bone does take place in this marrow, after the operation, is well known, especially from cases of excision of the knee. The bones left in this condition of atrophy soon acquired sufficient firmness to bear the weight of the body. We must, therefore, not be misled by this atrophic appearance, into removing more of the bone than we should do, if we saw firm osseous substance before us. The limb of the patient, whom I have just shown you, is now, one and a-half years since excision was made, strong enough to bear considerable weight.

Finally, I wish to mention a case where the operation was performed too late.

*Case 8.*—Patient was a boy of 12 years, pale and lean. There were several fistulæ, which discharged considerably; the leg was in a position of adduction and flexion. The disease had lasted four years. There was no fever, no tuberculosis of the lungs, no albuminuria. Patient had been in bed for over a year, and his appetite was exceedingly poor. Excision was made, unaccompanied by any complicating accident. There was no unusual vomiting subsequent to the narcosis, nor were any signs of carbolic acid poisoning to be detected in the urine. But patient gradually sank after the operation. His pulse remained 100; his temperature never rose above normal. Eleven days after the operation he died. The autopsy revealed no disease of any of the internal organs, no tuberculosis, no amyloid degenerations. But the whole body was in a condition of extreme anæmia. The wound had not suppurated, and was apparently on the way to healing by first intention.

Before concluding my remarks on hip operations, I wish to repeat that excision of the hip joint, in a chronic case of hip disease, and performed with observation of antiseptic precautions, is by no means a dangerous operation. The hæmorrhage is trifling, and the shock moderate. The danger of former times, those of a supervening septicæmia, or, as it is often called, especially by English writers, of exhaustion or exhaustive suppuration, it is at the present time in the power of the surgeon to exclude almost to a certainty. Fear of cutting short the patient's life is, consequently, no longer a strong reason for postponing the operation. On the other hand, if the operation be deferred too long, that is till septicæmia has established itself, the patient will die in spite of the operation. The main question to be decided in early operations does not refer to the life of the patient, but to the removal of living bone, which, later on, might become serviceable in supporting the body. Gouging out of the hip joint is technically impracticable; the attempts made have not given satisfactory results. Partial excisions, excisions of the diseased head alone, leaving part of the neck of the femur and the trochanter in place, have been tried, but again abandoned. As complete excision, through or below the great trochanter, is thus the only advisable form of the operation, the question, when should we operate? still presents the greatest difficulties in the solution of the whole problem.

*Excision of the Knee Joint.* What was said of the tuberculous infection when speaking of the hip joint, of course finds application here also. The results obtained by antiseptic surgery of the knee are excellent. The wounds heal speedily, and the danger to life is small. The death-rate has been reduced from about 30 per cent. to almost a trifling per centage. In a series of thirty-two excisions of the knee, made by Volkmann, there occurred not one death attributable to the operation. In excising a knee joint we have an additional object in view, which plays no part in operations upon the hip, namely, we try to make the cut surfaces of the femur and tibia grow together and to produce a solid anchylosis. The surfaces of the two bones should be cut in such a way as to secure a good position of the leg afterwards; they can easily be kept in apposition by means of wires, pins or nails. In a number of cases the bones unite in the course of a few weeks, the time in which we expect to get a union in cases of subcutaneous fractures.

Such a case is that of

*Case 1.*—James Carr, 15 years old, entered Cook County Hospital in July, 1879. He suffered from tuberculous arthritis of two years' standing. The leg was flexed to a right angle, the region of the joint was swollen and tender, and on each side there were scars from closed sinuses consequent upon abscesses. Excision was performed August 6. In three weeks a solid osseous union had taken place; in five weeks he was up and walked about with crutches. Ten weeks after the operation he was able to walk without the use of either crutch or cane. In the course of the following half year, tubercular abscesses and sinuses formed in different places in the cicatrix of the excision wound. Scraping and iodoform treatment caused these abscesses to close up definitively; their formation never interfered with the solid osseous union of the two bones.

There is now a solid anchylosis in a straight position; he is able to walk all day long, and perform any kind of work, without having any pain in the leg whatever.*

* This case was published in extenso in a former paper by me, printed in the *Chicago Medical Journal and Examiner*, in July, 1880.

This case, furthermore, illustrates that a secondary local growth of tubercles in and around the cicatrix does not necessarily destroy the effects of the operation, as far as a permanent cure and perfect usefulness of the limb are concerned.

In excisions of the knee joint, where alone we desire to obtain a good, solid anchylosis, we encounter a peculiar difficulty, and that is, that we by no means always get a good, solid anchylosis. For reasons not well understood, the bones do not grow together, but are held in union by more or less ligamentous tissue. This seems to happen more frequently in children than in adults. In these cases it does not always become apparent at once that the function of the limb will be destroyed. A shorter or longer time after the operation the the limb, which at first was in a good straight position, has become a little retracted or flexed, the degree of flexion grows worse, till finally a contracture is developed. In some instances this contracture will begin to come on during the early part of the convalescence; in others, the patient will get up, walk about with an apparently solid anchylosis, the leg will be in good position, and we may congratulate ourselves upon the good results of the operation. But it is too early. One, two, or three years later the contracture of the knee may begin. This contracture may, to a certain extent, be combated by immobilizing apparatus. Our patient should always be made aware of the probability of its occurrence. In adults, immobilizing apparatus put on timely, that is, as soon as any liability to contracture becomes apparent, may prevent the contracture from developing to such a degree as to interfere very much with the use of the limb. The matter is difficult in cases of small children. Not only is the liability to contracture here greater, but all experienced surgeons know that it is difficult and oftentimes altogether impossible to keep immobilizing bandages or apparatus on these little patientss for a sufficient length of time, and with a sufficient degree of immobility to prevent secondary contractures from coming on. Sometimes the apparatus produces pain, and cannot be borne on that account. Extension by weight and pulleys cannot be continued sufficiently long; for these little ones will get up and walk about. Small children cannot be taught to use crutches as efficiently as older ones can. They will step on the foot unless prevented therefrom by severe pain. The consequence of all this is, that in these cases nothing can be done to ward off the contracture. One or two years after the operation we often find the leg forming a rectangle with the thigh; the malposition is as bad as it was before the excision. There is, however, this difference between the two conditions that now the white swelling or tuberculosis is gone, the fistulæ have healed, and the joint is neither tender nor painful. The only but great trouble that remains is, that the limb is almost useless for walking purposes.

I have two cases in point here, and will show them to you

*Case 2.*—William Kane, 9 years old, was admitted to the Cook County Hospital, Sept. 6, 1880. His family history told of no cases of tuberculosis; he had two brothers and two sisters living and healthy. Two and a half years previous to his admission he had jumped off an ice-box and injured his knee. Soon afterwards the knee became swollen and painful. After hot applications had been made for two weeks the swelling was lanced and exit given to half a pint of matter. Poultices were then applied and the discharge continued. Three months later another abscess was lanced on the inner side of the knee and 5-6 ounces of pus evacuated. During the following four months several new abscesses formed along the tibia. He further stated that there had been eleven sinuses, and that at one time they were all discharging. Those on the leg closed in May, 1880. Patient was very low a year before the date of his admission; his death was then expected every hour, and he wasted away to such an extent that, as his father said, nothing seemed to be left of him but skin and bones. In April, 1880, his health began to improve, and some of the sinuses closed. At the time of his admission his general health was fair; he was fairly well nourished, had a good appetite and slept well. The right knee presented a deformity, the tibia being dislocated backwards, thereby producing an eversion of the foot. The anterior surface of the knee showed a running fistula. Along the inner side of the leg the marks of several closed sinuses were to be seen. The leg was drawn up almost to a right angle, and there was but a minimum of motion left in the joint. Pressure upon the knee was not painful. Patient managed to walk about with the aid of crutches.

Sept. 17. Excision of the knee-joint. A horseshoe-shaped incision was made below the patella and the patella removed. The condyles of the femur were sawed off close to the line of the joint. The external condyle had almost entirely disappeared. The internal condyle formed the inner termination of the sinus which opened at the anterior inner part of the knee. One sixth of an inch of the tibia was removed and the sawn surfaces of the femur and tibia united and held together with two pieces of silver wire. Drainage-tubes were introduced and placed before and behind the bone, the wound was closed and an antiseptic dressing applied. The leg was placed in a straight tin splint with side-doors, and then suspended from a frame.

Sept. 19. Temperature, 102°.

Sept. 22. The leg was dressed; a moderate discharge of matter was found.

Sept. 24. Temperature 101.5°. The wound was dressed daily; the discharge had a perceptible odor.

Sept. 25. An abscess which had formed near the head of the fibula, was opened and gave exit to ½ ounce of pus. In the course of the following week several abscesses formed along the tibia at the places where the old sinuses had been. They were opened and drained one after the other. In the beginning of October the temperature returned to normal. Two weeks later the discharge stopped, and the femur and tibia had become somewhat united. About the middle of October the wound and all the sinuses had healed up, and the splint was removed.

Patient was dismissed with the advice to use crutches in walking and to keep the foot of the diseased leg from the ground. The bony union had not yet grown sufficiently firm and the limb showed some tendency to flexion.

At the present time the leg is flexed in the knee to a right angle; there is no sinus, no swelling, no tenderness; but a solid osseous union between the cut surfaces of the bones did not take place, and we detect a slight mobility on making forcible flexion and extension.

*Case 3.*—John Hostetter, aged six, fell when one year old. A tuberculosis of the knee joint ensued, and, in the course of the following three years, abscesses formed, and kept discharging. A contraction beyond a right angle, together with dislocation of the head of the tibia backwards on to the posterior surface of the condyles of the femur gradually developed. Two years ago there was an ulcerated surface over the internal condyle of the femur of over an inch in diameter, the bottom of which was formed by the denuded bone. At that time, two years ago, I made the excision; wound and sinuses healed in a couple of months. No perfect osseous union took place. In the course of half a year a contraction to a right angle had been formed. Now there is neither tenderness nor swelling in the region of the joint, nor any open sinuses, but the leg is flexed to a right angle. He limps about without having any pain, but is greatly inconvenienced by the deformity.

In these two little patients, further surgical procedures, forcible stretching or nei-form osteotomy, will be necessary to procure a straight position of the limbs. In that respect the operations may be said to have failed of accomplishing their objects. It would, however, be going a little too far to assert that in these two cases the operations were entirely useless. In both cases the local tuberculous inflammation, with its attending pain and debilitating effects on the general health, was successfully checked. (Besides, as these patients are always able to use the limb somewhat, the atrophy of inactivity, the retarded growth of the leg and foot, is considerably helped by the operation. This is the more important the younger the child is, as in young children growth is most energetic.)

Volkmann proposed a new method of operating in these cases. He makes a transverse incision, and then bisects the patella. Primary union of the patella may always be expected to take place in an aseptic case. Whether such operations will be followed by contractions or not is a question which the future will have to answer. Older statistics, to be sure, show a greater mortality in cases where the patella was allowed to remain than where it was removed. But this does not constitute an objection to Volkmann's operation, for, armed as we are with the antiseptic method, we no longer fear such dangers as might threaten from a remaining patella.

As we have seen, there is one great imperfection in the results of excision of tuberculous knee joints. Nevertheless, I feel justified in asserting that, in almost all cases of white swelling or tuberculosis of the knee, an attempt should be made to save the limb by conservative surgery, by gouging out the tuberculous joint, or by an excision of the same, and that amputation of a limb in a case of white swelling, without such previous attempt, should become one of the rarest exceptions.

214 East Ohio Street, Chicago.

